# Supplementary material for: A Proteomic Approach for Understanding the Mechanisms of Delayed Corneal Wound Healing in Diabetic Keratopathy Using Diabetic Model Rat
Source: Int J Mol Sci. 2018 Nov 18;19(11):3635. doi: 10.3390/ijms19113635 (PMC6274742; doi:10.3390/ijms19113635)
Supplement: Supplementary file 1 [file ijms-19-03635-s001.zip › ijms-385001 supplementary/Supplementary Figure.pptx]

## Slide 1
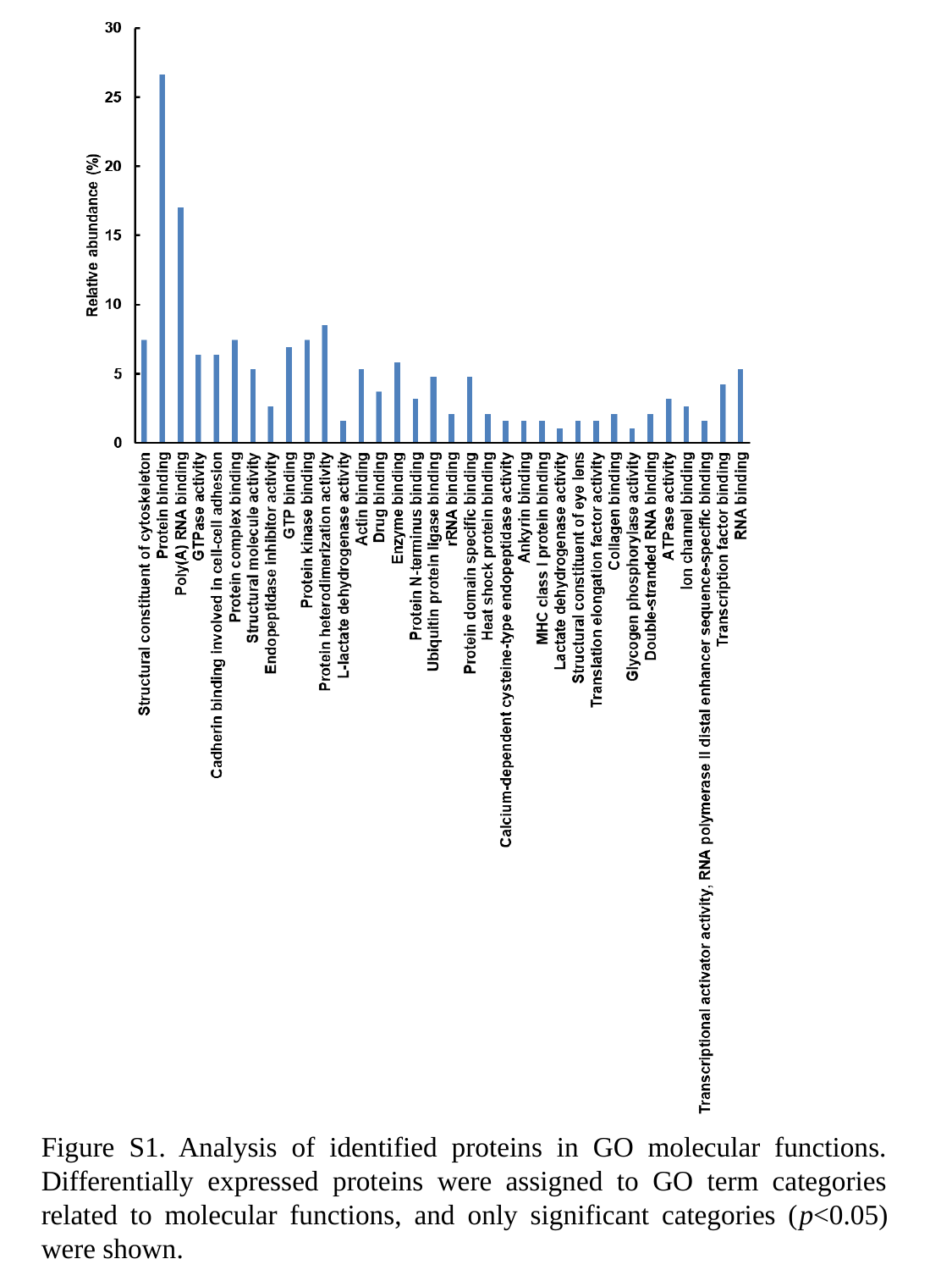

Figure S1. Analysis of identified proteins in GO molecular functions. Differentially expressed proteins were assigned to GO term categories related to molecular functions, and only significant categories (p<0.05) were shown.

## Slide 2
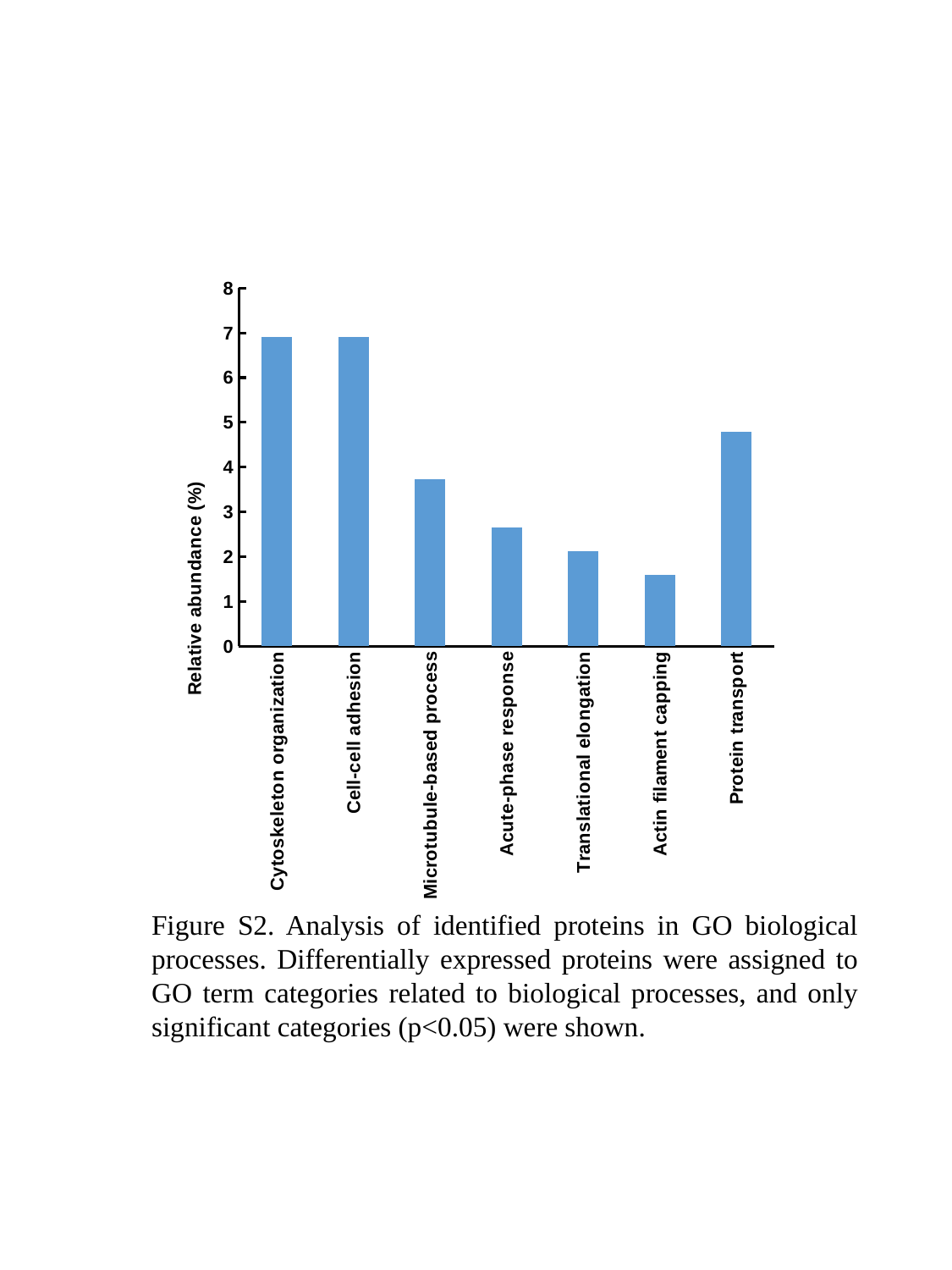

### Chart
| Category | |
|---|---|
| Cytoskeleton organization | 6.91489361702127 |
| Cell-cell adhesion | 6.91489361702127 |
| Microtubule-based process | 3.72340425531914 |
| Acute-phase response | 2.6595744680851 |
| Translational elongation | 2.12765957446808 |
| Actin filament capping | 1.59574468085106 |
| Protein transport | 4.78723404255319 |Figure S2. Analysis of identified proteins in GO biological processes. Differentially expressed proteins were assigned to GO term categories related to biological processes, and only significant categories (p<0.05) were shown.

## Slide 3
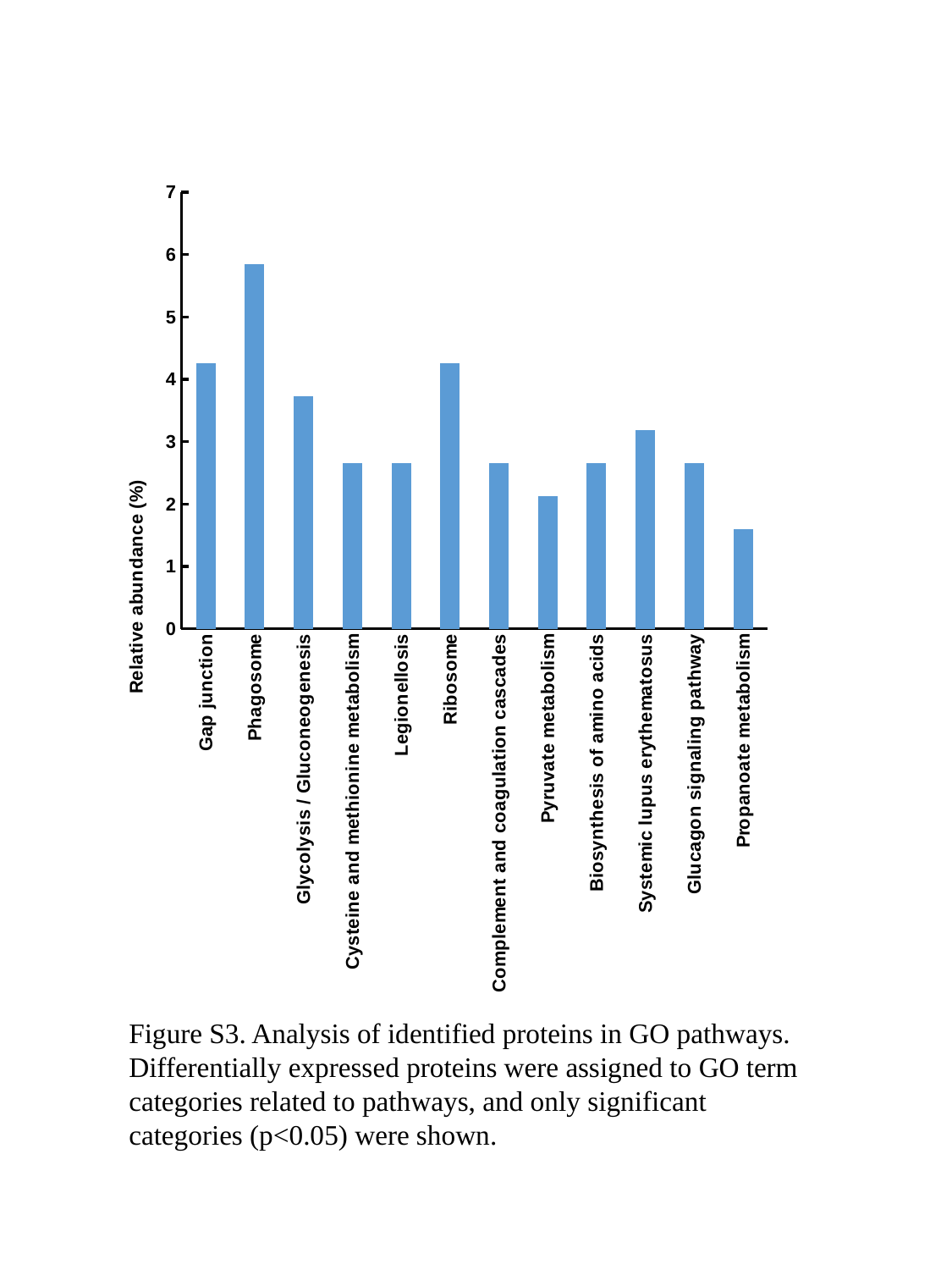

### Chart
| Category | |
|---|---|
| Gap junction | 4.25531914893617 |
| Phagosome | 5.85106382978723 |
| Glycolysis / Gluconeogenesis | 3.72340425531914 |
| Cysteine and methionine metabolism | 2.6595744680851 |
| Legionellosis | 2.6595744680851 |
| Ribosome | 4.25531914893617 |
| Complement and coagulation cascades | 2.6595744680851 |
| Pyruvate metabolism | 2.12765957446808 |
| Biosynthesis of amino acids | 2.6595744680851 |
| Systemic lupus erythematosus | 3.19148936170212 |
| Glucagon signaling pathway | 2.6595744680851 |
| Propanoate metabolism | 1.59574468085106 |
### Chart
| Category |
|---|Figure S3. Analysis of identified proteins in GO pathways. Differentially expressed proteins were assigned to GO term categories related to pathways, and only significant categories (p<0.05) were shown.
