# Supplementary material for: A Proteomic Approach for Understanding the Mechanisms of Delayed Corneal Wound Healing in Diabetic Keratopathy Using Diabetic Model Rat
Source: Int J Mol Sci. 2018 Nov 18;19(11):3635. doi: 10.3390/ijms19113635 (PMC6274742; doi:10.3390/ijms19113635)
Supplement: Supplementary file 1 [file ijms-19-03635-s001.zip › ijms-385001 supplementary/Supplementary Table S1.docx]

Table S1. Proteins differentially expressed (≥2-fold) in cornea of STZ rats.

| No. | ID | Accession number and description | | Number of amino acids | Spectral counting | | |
| --- | --- | --- | --- | --- | --- | --- | --- |
|  |  |  |  |  | Normal | STZ | Fold change (Rsc) |
| 1 | K2C8_RAT | Q10758 | Keratin, type II cytoskeletal 8 | 483 | 28 | 0 | -4.304832174 |
| 2 | CRGF_RAT | P10068 | Gamma-crystallin F | 174 | 15 | 0 | -3.447924425 |
| 3 | PLEC_RAT | P30427 | Plectin | 4687 | 6 | 0 | -2.277400646 |
| 4 | TBB5_RAT | P69897 | Tubulin beta-5 chain | 444 | 16 | 2 | -2.154623931 |
| 5 | K1C17_RAT | Q6IFU8 | Keratin, type I cytoskeletal 17 | 433 | 5 | 0 | -2.062595565 |
| 6 | EF2_RAT | P05197 | Elongation factor 2 | 858 | 5 | 0 | -2.062595565 |
| 7 | TBB4B_RAT | Q6P9T8 | Tubulin beta-4B chain | 445 | 5 | 0 | -2.062595565 |
| 8 | K1C42_RAT | Q6IFU7 | Keratin, type I cytoskeletal 42 | 452 | 14 | 2 | -1.975470138 |
| 9 | H2AJ_RAT | A9UMV8 | Histone H2A.J | 129 | 4 | 0 | -1.810376843 |
| 10 | H2A1C_RAT | P0C169 | Histone H2A type 1-C | 130 | 8 | 1 | -1.781423348 |
| 11 | EF1A2_RAT | P62632 | Elongation factor 1-alpha 2 | 463 | 11 | 2 | -1.657395058 |
| 12 | NFH_RAT | P16884 | Neurofilament heavy polypeptide | 1072 | 7 | 1 | -1.615683184 |
| 13 | K2C73_RAT | Q6IG03 | Keratin, type II cytoskeletal 73 | 553 | 14 | 3 | -1.587631817 |
| 14 | PPIA_RAT | P10111 | Peptidyl-prolyl cis-trans isomerase A | 164 | 17 | 4 | -1.543099296 |
| 15 | K1C13_RAT | Q6IFV4 | Keratin, type I cytoskeletal 13 | 438 | 3 | 0 | -1.504842626 |
| 16 | HNRPK_RAT | P61980 | Heterogeneous nuclear ribonucleoprotein K | 463 | 3 | 0 | -1.504842626 |
| 17 | MDHM_RAT | P04636 | Malate dehydrogenase, mitochondrial | 338 | 3 | 0 | -1.504842626 |
| 18 | PRDX2_RAT | P35704 | Peroxiredoxin-2 | 198 | 3 | 0 | -1.504842626 |
| 19 | ARF4_RAT | P61751 | ADP-ribosylation factor 4 | 180 | 3 | 0 | -1.504842626 |
| 20 | A1AG_RAT | P02764 | Alpha-1-acid glycoprotein | 205 | 3 | 0 | -1.504842626 |
| 21 | TBB2A_RAT | P85108 | Tubulin beta-2A chain | 445 | 19 | 5 | -1.442138472 |
| 22 | K1C10_RAT | Q6IFW6 | Keratin, type I cytoskeletal 10 | 526 | 6 | 1 | -1.428589463 |
| 23 | TBA1B_RAT | Q6P9V9 | Tubulin alpha-1B chain | 451 | 6 | 1 | -1.428589463 |
| 24 | TBB3_RAT | Q4QRB4 | Tubulin beta-3 chain | 450 | 8 | 2 | -1.250093895 |
| 25 | TBA1A_RAT | P68370 | Tubulin alpha-1A chain | 451 | 5 | 1 | -1.213784382 |
| 26 | K1C15_RAT | Q6IFV3 | Keratin, type I cytoskeletal 15 | 447 | 13 | 4 | -1.183431403 |
| 27 | EF1A1_RAT | P62630 | Elongation factor 1-alpha 1 | 462 | 15 | 5 | -1.121920342 |
| 28 | TBA3_RAT | Q68FR8 | Tubulin alpha-3 chain | 450 | 2 | 0 | -1.117140188 |
| 29 | GELS_RAT | Q68FP1 | Gelsolin | 780 | 2 | 0 | -1.117140188 |
| 30 | RLA2_RAT | P02401 | 60S acidic ribosomal protein P2 | 115 | 2 | 0 | -1.117140188 |
| 31 | COF1_RAT | P45592 | Cofilin-1 | 166 | 2 | 0 | -1.117140188 |
| 32 | PRDX1_RAT | Q63716 | Peroxiredoxin-1 | 199 | 2 | 0 | -1.117140188 |
| 33 | CLH1_RAT | P11442 | Clathrin heavy chain 1 | 1675 | 2 | 0 | -1.117140188 |
| 34 | PPME1_RAT | Q4FZT2 | Protein phosphatase methylesterase 1 | 386 | 2 | 0 | -1.117140188 |
| 35 | H2AY_RAT | Q02874 | Core histone macro-H2A.1 | 371 | 2 | 0 | -1.117140188 |
| 36 | RS18_RAT | P62271 | 40S ribosomal protein S18 | 152 | 2 | 0 | -1.117140188 |
| 37 | 1433E_RAT | P62260 | 14-3-3 protein epsilon | 255 | 2 | 0 | -1.117140188 |
| 38 | HSP7C_RAT | P63018 | Heat shock cognate 71 kDa protein | 646 | 2 | 0 | -1.117140188 |
| 39 | PLBL2_RAT | Q4QQW8 | Putative phospholipase B-like 2 | 585 | 2 | 0 | -1.117140188 |
| 40 | CAN2_RAT | Q07009 | Calpain-2 catalytic subunit | 700 | 2 | 0 | -1.117140188 |
| 41 | PTGR2_RAT | Q5BK81 | Prostaglandin reductase 2 | 351 | 2 | 0 | -1.117140188 |
| 42 | LIN7B_RAT | Q9Z252 | Protein lin-7 homolog B | 207 | 2 | 0 | -1.117140188 |
| 43 | RS15A_RAT | P62246 | 40S ribosomal protein S15a | 130 | 2 | 0 | -1.117140188 |
| 44 | CACB3_RAT | P54287 | Voltage-dependent L-type calcium channel subunit beta-3 | 484 | 2 | 0 | -1.117140188 |
| 45 | TBKB1_RAT | Q6DG50 | TANK-binding kinase 1-binding protein 1 | 613 | 2 | 0 | -1.117140188 |
| 46 | MAGI3_RAT | Q9JK71 | Membrane-associated guanylate kinase, WW and PDZ domain-containing protein 3 | 1470 | 2 | 0 | -1.117140188 |
| 47 | KCNJ3_RAT | P63251 | G protein-activated inward rectifier potassium channel 1 | 501 | 2 | 0 | -1.117140188 |
| 48 | CRGB_RAT | P10066 | Gamma-crystallin B | 175 | 18 | 32 | 1.065247274 |
| 49 | GBLP_RAT | P63245 | Guanine nucleotide-binding protein subunit beta-2-like 1 | 317 | 0 | 1 | 1.111540289 |
| 50 | PA2G4_RAT | Q6AYD3 | Proliferation-associated protein 2G4 | 394 | 0 | 1 | 1.111540289 |
| 51 | OTUB1_RAT | B2RYG6 | Ubiquitin thioesterase OTUB1 | 271 | 0 | 1 | 1.111540289 |
| 52 | LDHB_RAT | P42123 | L-lactate dehydrogenase B chain | 334 | 0 | 1 | 1.111540289 |
| 53 | LDHC_RAT | P19629 | L-lactate dehydrogenase C chain | 332 | 0 | 1 | 1.111540289 |
| 54 | GLOD4_RAT | Q5I0D1 | Glyoxalase domain-containing protein 4 | 298 | 0 | 1 | 1.111540289 |
| 55 | TCP4_RAT | Q63396 | Activated RNA polymerase II transcriptional coactivator p15 | 127 | 0 | 1 | 1.111540289 |
| 56 | EST1C_RAT | P10959 | Carboxylesterase 1C | 549 | 0 | 1 | 1.111540289 |
| 57 | LCA5_RAT | Q5U2Y9 | Lebercilin | 727 | 0 | 1 | 1.111540289 |
| 58 | PHAR1_RAT | P62024 | Phosphatase and actin regulator 1 | 580 | 0 | 1 | 1.111540289 |
| 59 | VDAC1_RAT | Q9Z2L0 | Voltage-dependent anion-selective channel protein 1 | 283 | 0 | 1 | 1.111540289 |
| 60 | TPIS_RAT | P48500 | Triosephosphate isomerase | 249 | 0 | 1 | 1.111540289 |
| 61 | GBB2_RAT | P54313 | Guanine nucleotide-binding protein G(I)/G(S)/G(T) subunit beta-2 | 340 | 0 | 1 | 1.111540289 |
| 62 | ZW10_RAT | Q4V8C2 | Centromere/kinetochore protein zw10 homolog | 777 | 0 | 1 | 1.111540289 |
| 63 | ABCC9_RAT | Q63563 | ATP-binding cassette sub-family C member 9 | 1545 | 0 | 1 | 1.111540289 |
| 64 | B3AT_RAT | P23562 | Band 3 anion transport protein | 927 | 0 | 1 | 1.111540289 |
| 65 | CCNF_RAT | Q8K4F8 | Cyclin-F | 780 | 0 | 1 | 1.111540289 |
| 66 | XKR7_RAT | Q5GH56 | XK-related protein 7 | 580 | 0 | 1 | 1.111540289 |
| 67 | CA131_RAT | Q3KRF3 | Uncharacterized protein C1orf131 homolog | 282 | 0 | 1 | 1.111540289 |
| 68 | SPTN2_RAT | Q9QWN8 | Spectrin beta chain, non-erythrocytic 2 | 2388 | 0 | 1 | 1.111540289 |
| 69 | CPNS1_RAT | Q64537 | Calpain small subunit 1 | 270 | 0 | 1 | 1.111540289 |
| 70 | CGRE1_RAT | P97586 | Cell growth regulator with EF hand domain protein 1 | 281 | 0 | 1 | 1.111540289 |
| 71 | GBP2_RAT | Q63663 | Interferon-induced guanylate-binding protein 2 | 589 | 0 | 1 | 1.111540289 |
| 72 | RL9_RAT | P17077 | 60S ribosomal protein L9 | 192 | 0 | 1 | 1.111540289 |
| 73 | ARHGP_RAT | Q6P720 | Rho guanine nucleotide exchange factor 25 | 579 | 0 | 1 | 1.111540289 |
| 74 | SUGT1_RAT | B0BN85 | Suppressor of G2 allele of SKP1 homolog | 336 | 0 | 1 | 1.111540289 |
| 75 | MROH7_RAT | A2RUW0 | Maestro heat-like repeat-containing protein family member 7 | 1122 | 0 | 1 | 1.111540289 |
| 76 | MEOX2_RAT | P39020 | Homeobox protein MOX-2 | 303 | 0 | 1 | 1.111540289 |
| 77 | GSTA1_RAT | P00502 | Glutathione S-transferase alpha-1 | 222 | 0 | 1 | 1.111540289 |
| 78 | LIX1L_RAT | Q5PQQ7 | LIX1-like protein | 338 | 0 | 1 | 1.111540289 |
| 79 | COQ9_RAT | Q68FT1 | Ubiquinone biosynthesis protein COQ9, mitochondrial | 312 | 0 | 1 | 1.111540289 |
| 80 | KCNQ3_RAT | O88944 | Potassium voltage-gated channel subfamily KQT member 3 | 873 | 0 | 1 | 1.111540289 |
| 81 | LRP2_RAT | P98158 | Low-density lipoprotein receptor-related protein 2 | 4660 | 0 | 1 | 1.111540289 |
| 82 | LMNB1_RAT | P70615 | Lamin-B1 | 587 | 0 | 1 | 1.111540289 |
| 83 | AIFM1_RAT | Q9JM53 | Apoptosis-inducing factor 1, mitochondrial | 612 | 0 | 1 | 1.111540289 |
| 84 | NCOA2_RAT | Q9WUI9 | Nuclear receptor coactivator 2 | 1465 | 0 | 1 | 1.111540289 |
| 85 | Z518A_RAT | Q499R0 | Zinc finger protein 518A | 1478 | 0 | 1 | 1.111540289 |
| 86 | MADD_RAT | O08873 | MAP kinase-activating death domain protein | 1602 | 0 | 1 | 1.111540289 |
| 87 | ENOB_RAT | P15429 | Beta-enolase | 434 | 0 | 1 | 1.111540289 |
| 88 | TSP4_RAT | P49744 | Thrombospondin-4 | 980 | 0 | 1 | 1.111540289 |
| 89 | GLNA_RAT | P09606 | Glutamine synthetase | 373 | 0 | 1 | 1.111540289 |
| 90 | CO1A2_RAT | P02466 | Collagen alpha-2(I) chain | 1372 | 0 | 1 | 1.111540289 |
| 91 | CCD93_RAT | Q5BJT7 | Coiled-coil domain-containing protein 93 | 629 | 0 | 1 | 1.111540289 |
| 92 | SYG_RAT | Q5I0G4 | Glycine--tRNA ligase (Fragment) | 637 | 0 | 1 | 1.111540289 |
| 93 | ZFP57_RAT | A0JPK3 | Zinc finger protein 57 | 406 | 0 | 1 | 1.111540289 |
| 94 | TTC29_RAT | Q6AYP3 | Tetratricopeptide repeat protein 29 | 471 | 0 | 1 | 1.111540289 |
| 95 | CNTN1_RAT | Q63198 | Contactin-1 | 1021 | 0 | 1 | 1.111540289 |
| 96 | TERA_RAT | P46462 | Transitional endoplasmic reticulum ATPase | 806 | 0 | 1 | 1.111540289 |
| 97 | RBM47_RAT | Q66H68 | RNA-binding protein 47 | 590 | 0 | 1 | 1.111540289 |
| 98 | MCES_RAT | Q5U2U7 | mRNA cap guanine-N7 methyltransferase | 461 | 0 | 1 | 1.111540289 |
| 99 | TM40L_RAT | A4F267 | Mitochondrial import receptor subunit TOM40B | 308 | 0 | 1 | 1.111540289 |
| 100 | GDNF_RAT | Q07731 | Glial cell line-derived neurotrophic factor | 211 | 0 | 1 | 1.111540289 |
| 101 | SRSF2_RAT | Q6PDU1 | Serine/arginine-rich splicing factor 2 | 221 | 0 | 1 | 1.111540289 |
| 102 | PDZ1I_RAT | Q923S2 | PDZK1-interacting protein 1 | 114 | 0 | 1 | 1.111540289 |
| 103 | MDGA1_RAT | P85171 | MAM domain-containing glycosylphosphatidylinositol anchor protein 1 | 956 | 0 | 1 | 1.111540289 |
| 104 | PLCE1_RAT | Q99P84 | 1-phosphatidylinositol 4,5-bisphosphate phosphodiesterase epsilon-1 | 2281 | 0 | 1 | 1.111540289 |
| 105 | PCLO_RAT | Q9JKS6 | Protein piccolo | 5085 | 0 | 1 | 1.111540289 |
| 106 | IDLC_RAT | Q4FZV3 | Axonemal dynein light intermediate polypeptide 1 | 258 | 0 | 1 | 1.111540289 |
| 107 | DYHC2_RAT | Q9JJ79 | Cytoplasmic dynein 2 heavy chain 1 | 4306 | 0 | 1 | 1.111540289 |
| 108 | ASHWN_RAT | Q5RJT0 | Ashwin | 232 | 0 | 1 | 1.111540289 |
| 109 | PYGL_RAT | P09811 | Glycogen phosphorylase, liver form | 850 | 0 | 1 | 1.111540289 |
| 110 | NPM_RAT | P13084 | Nucleophosmin | 292 | 0 | 1 | 1.111540289 |
| 111 | SMCA4_RAT | Q8K1P7 | Transcription activator BRG1 | 1613 | 0 | 1 | 1.111540289 |
| 112 | HP1B3_RAT | Q6P747 | Heterochromatin protein 1-binding protein 3 | 553 | 0 | 1 | 1.111540289 |
| 113 | CU059_RAT | Q5U3Z0 | UPF0769 protein C21orf59 homolog | 290 | 0 | 1 | 1.111540289 |
| 114 | PHOCN_RAT | Q9QYW3 | MOB-like protein phocein | 225 | 0 | 1 | 1.111540289 |
| 115 | RL18A_RAT | P62718 | 60S ribosomal protein L18a | 176 | 0 | 1 | 1.111540289 |
| 116 | ROCK1_RAT | Q63644 | Rho-associated protein kinase 1 | 1369 | 0 | 1 | 1.111540289 |
| 117 | IF4A2_RAT | Q5RKI1 | Eukaryotic initiation factor 4A-II | 407 | 0 | 1 | 1.111540289 |
| 118 | ARPC2_RAT | P85970 | Actin-related protein 2/3 complex subunit 2 | 300 | 0 | 1 | 1.111540289 |
| 119 | GSTT1_RAT | Q01579 | Glutathione S-transferase theta-1 | 240 | 0 | 1 | 1.111540289 |
| 120 | S35E1_RAT | P0C6B1 | Solute carrier family 35 member E1 | 409 | 0 | 1 | 1.111540289 |
| 121 | VPS25_RAT | P0C0A1 | Vacuolar protein-sorting-associated protein 25 | 176 | 0 | 1 | 1.111540289 |
| 122 | MUG1_RAT | Q03626 | Murinoglobulin-1 | 1487 | 0 | 1 | 1.111540289 |
| 123 | MAP1A_RAT | P34926 | Microtubule-associated protein 1A | 2774 | 0 | 1 | 1.111540289 |
| 124 | CP4F4_RAT | P51869 | Cytochrome P450 4F4 | 522 | 0 | 1 | 1.111540289 |
| 125 | ZG16_RAT | Q8CJD3 | Zymogen granule membrane protein 16 | 167 | 0 | 1 | 1.111540289 |
| 126 | NIBL1_RAT | B4F7E8 | Niban-like protein 1 | 747 | 0 | 1 | 1.111540289 |
| 127 | CAD17_RAT | P55281 | Cadherin-17 | 827 | 0 | 1 | 1.111540289 |
| 128 | PANX2_RAT | P60571 | Pannexin-2 | 674 | 0 | 1 | 1.111540289 |
| 129 | IF172_RAT | Q9JKU3 | Intraflagellar transport protein 172 homolog | 1749 | 0 | 1 | 1.111540289 |
| 130 | UBF1_RAT | P25977 | Nucleolar transcription factor 1 | 764 | 0 | 1 | 1.111540289 |
| 131 | CDX1_RAT | Q05095 | Homeobox protein CDX-1 (Fragment) | 123 | 0 | 1 | 1.111540289 |
| 132 | NAA35_RAT | Q6DKG0 | N-alpha-acetyltransferase 35, NatC auxiliary subunit | 725 | 0 | 1 | 1.111540289 |
| 133 | NOL10_RAT | Q66H99 | Nucleolar protein 10 | 688 | 0 | 1 | 1.111540289 |
| 134 | TBA8_RAT | Q6AY56 | Tubulin alpha-8 chain | 449 | 0 | 1 | 1.111540289 |
| 135 | RS3_RAT | P62909 | 40S ribosomal protein S3 | 243 | 0 | 1 | 1.111540289 |
| 136 | TACD2_RAT | Q6P9Z6 | Tumor-associated calcium signal transducer 2 | 317 | 0 | 1 | 1.111540289 |
| 137 | ACE2_RAT | Q5EGZ1 | Angiotensin-converting enzyme 2 | 805 | 0 | 1 | 1.111540289 |
| 138 | ESR1_RAT | P06211 | Estrogen receptor | 600 | 0 | 1 | 1.111540289 |
| 139 | SIA7C_RAT | Q64686 | Alpha-N-acetylgalactosaminide alpha-2,6-sialyltransferase 3 | 305 | 0 | 1 | 1.111540289 |
| 140 | DIXC1_RAT | Q2VUH7 | Dixin | 674 | 0 | 1 | 1.111540289 |
| 141 | AT2A2_RAT | P11507 | Sarcoplasmic/endoplasmic reticulum calcium ATPase 2 | 1043 | 0 | 1 | 1.111540289 |
| 142 | DPOA2_RAT | O89043 | DNA polymerase alpha subunit B | 600 | 0 | 1 | 1.111540289 |
| 143 | ERBB3_RAT | Q62799 | Receptor tyrosine-protein kinase erbB-3 | 1339 | 0 | 1 | 1.111540289 |
| 144 | ACHB3_RAT | P12391 | Neuronal acetylcholine receptor subunit beta-3 | 464 | 0 | 1 | 1.111540289 |
| 145 | MECR_RAT | Q9Z311 | Trans-2-enoyl-CoA reductase, mitochondrial | 373 | 0 | 1 | 1.111540289 |
| 146 | P3H1_RAT | Q9R1J8 | Prolyl 3-hydroxylase 1 | 728 | 0 | 1 | 1.111540289 |
| 147 | SMK_RAT | A1A5Q6 | Sperm motility kinase | 654 | 0 | 1 | 1.111540289 |
| 148 | LYPA2_RAT | Q9QYL8 | Acyl-protein thioesterase 2 | 231 | 0 | 1 | 1.111540289 |
| 149 | TCAL7_RAT | D3ZT37 | Transcription elongation factor A protein-like 7 | 98 | 0 | 1 | 1.111540289 |
| 150 | HRH4_RAT | Q91ZY1 | Histamine H4 receptor | 391 | 0 | 1 | 1.111540289 |
| 151 | PO210_RAT | P11654 | Nuclear pore membrane glycoprotein 210 | 1886 | 0 | 1 | 1.111540289 |
| 152 | RGS19_RAT | O70521 | Regulator of G-protein signaling 19 | 216 | 0 | 1 | 1.111540289 |
| 153 | EDC4_RAT | Q3ZAV8 | Enhancer of mRNA-decapping protein 4 | 1407 | 0 | 1 | 1.111540289 |
| 154 | DPCR1_RAT | Q6MG22 | Diffuse panbronchiolitis critical region protein 1 homolog | 470 | 0 | 1 | 1.111540289 |
| 155 | UBP54_RAT | Q6IE24 | Inactive ubiquitin carboxyl-terminal hydrolase 54 | 1588 | 0 | 1 | 1.111540289 |
| 156 | PEF1_RAT | Q641Z8 | Peflin | 283 | 0 | 1 | 1.111540289 |
| 157 | GRIFN_RAT | O88644 | Grifin | 144 | 1 | 3 | 1.182032481 |
| 158 | RS19_RAT | P17074 | 40S ribosomal protein S19 | 145 | 1 | 3 | 1.182032481 |
| 159 | LDHA_RAT | P04642 | L-lactate dehydrogenase A chain | 332 | 2 | 5 | 1.208863895 |
| 160 | ENOA_RAT | P04764 | Alpha-enolase | 434 | 6 | 13 | 1.24418289 |
| 161 | LUM_RAT | P51886 | Lumican | 338 | 5 | 13 | 1.458987971 |
| 162 | PRELP_RAT | Q9EQP5 | Prolargin | 377 | 1 | 4 | 1.48770272 |
| 163 | CO3_RAT | P01026 | Complement C3 | 1663 | 1 | 4 | 1.48770272 |
| 164 | FABP5_RAT | P55053 | Fatty acid-binding protein, epidermal | 135 | 1 | 4 | 1.48770272 |
| 165 | A2MG_RAT | P06238 | Alpha-2-macroglobulin | 1472 | 9 | 23 | 1.517815975 |
| 166 | CRBS_RAT | P0C5E9 | Beta-crystallin S | 178 | 7 | 20 | 1.63934198 |
| 167 | GDIB_RAT | P50399 | Rab GDP dissociation inhibitor beta | 445 | 0 | 2 | 1.642869743 |
| 168 | RL11_RAT | P62914 | 60S ribosomal protein L11 | 178 | 0 | 2 | 1.642869743 |
| 169 | PDIA3_RAT | P11598 | Protein disulfide-isomerase A3 | 505 | 0 | 2 | 1.642869743 |
| 170 | CO4_RAT | P08649 | Complement C4 | 1737 | 0 | 2 | 1.642869743 |
| 171 | RS27A_RAT | P62982 | Ubiquitin-40S ribosomal protein S27a | 156 | 0 | 2 | 1.642869743 |
| 172 | METH_RAT | Q9Z2Q4 | Methionine synthase | 1253 | 0 | 2 | 1.642869743 |
| 173 | AL3B1_RAT | Q5XI42 | Aldehyde dehydrogenase family 3 member B1 | 468 | 0 | 2 | 1.642869743 |
| 174 | PON2_RAT | Q6AXM8 | Serum paraoxonase/arylesterase 2 | 354 | 0 | 2 | 1.642869743 |
| 175 | ACTS_RAT | P68136 | Actin, alpha skeletal muscle | 377 | 0 | 2 | 1.642869743 |
| 176 | PGS1_RAT | P47853 | Biglycan | 369 | 1 | 5 | 1.740057607 |
| 177 | FIBB_RAT | P14480 | Fibrinogen beta chain | 479 | 0 | 3 | 2.030708063 |
| 178 | ACTN1_RAT | Q9Z1P2 | Alpha-actinin-1 | 892 | 0 | 3 | 2.030708063 |
| 179 | PYGB_RAT | P53534 | Glycogen phosphorylase, brain form (Fragment) | 838 | 0 | 3 | 2.030708063 |
| 180 | SPTN1_RAT | P16086 | Spectrin alpha chain, non-erythrocytic 1 | 2472 | 0 | 3 | 2.030708063 |
| 181 | ABCA7_RAT | Q7TNJ2 | ATP-binding cassette sub-family A member 7 | 2170 | 0 | 3 | 2.030708063 |
| 182 | PGS2_RAT | Q01129 | Decorin | 354 | 2 | 10 | 2.060948338 |
| 183 | DEST_RAT | Q7M0E3 | Destrin | 165 | 1 | 7 | 2.142229161 |
| 184 | COTL1_RAT | B0BNA5 | Coactosin-like protein | 142 | 0 | 4 | 2.336378302 |
| 185 | TCTP_RAT | P63029 | Translationally-controlled tumor protein | 172 | 0 | 4 | 2.336378302 |
| 186 | H31_RAT | Q6LED0 | Histone H3.1 | 136 | 0 | 4 | 2.336378302 |
| 187 | A1AT_RAT | P17475 | Alpha-1-antiproteinase | 411 | 0 | 8 | 3.156781497 |
| 188 | IGG2A_RAT | P20760 | Ig gamma-2A chain C region | 322 | 0 | 12 | 3.678523253 |

Expression levels of these 188 proteins were more than 2-fold higher or lower in cornea of STZ treatment rat compared to cornea of normal rat samples.
